# Reporting and Grading of Complications in Urological Surgery: Current Trends and Future Perspectives

**DOI:** 10.5152/tud.2024.24050

**Published:** 2024-05-01

**Authors:** Stamatios Katsimperis, Themistoklis Bellos, Ioannis Manolitsis, Ioannis Kyriazis, Panagiotis Angelopoulos, Panagiotis Neophytou, Sotirios Kapsalos, Nikolaos Kostakopoulos, Lazaros Tzelves, Ioannis Varkarakis, Athanasios Papatsoris, Andreas Skolarikos, Iraklis Mitsogiannis

**Affiliations:** 1 Department of Urology, University of Athens, Sismanogleio General Hospital, Athens, Greece; 2 Department of Urology, Metropolitan General, Athens, Greece

**Keywords:** Complications, complication reporting, urology, surgery, complication grading, surgical complications

## Abstract

There has been a growing need for enhancements in healthcare delivery, especially for the improvement of surgical outcomes. Therefore, implementing consistent reporting of complications enables the evaluation of data quality and facilitates its comparison. There are currently many available reporting and grading systems each with its own set of benefits and drawbacks. In this comprehensive review, we tried to present and assess each of them by demonstrating their criteria and their strong and weak points. To sum up, it seems that there is a need for developing a new reporting and categorization system for complications that are specific to urology.

**Main Points**

- Clavien-Dindo Classification (CDC) system does not meet the needs of today’s complication grading.
- New reporting and grading systems have emerged, such as comprehensive complication index, the Intraoperative Adverse Incident Classification by EAU, the EAU Guidelines Panel criteria for reporting complications, and many more.
- There is still a need for developing a new reporting and categorization system for complications that is specific to urology.

## Introduction

All surgical procedures carry a risk of intra- or postoperative complications, which may exert significant financial pressure on healthcare systems.^1^ Therefore, systematic and high-quality documentation of procedure-related adverse events, along with appropriate preoperative workup, is of utmost importance. This may aid in identifying systematic errors in our surgical practice and significantly enhance patient care. Unfortunately, inaccuracies in reporting complications are common amongst surgeons.^2^ Thus, a precise and reproducible classification of complications is crucial. This can only be achieved using a dependable and verified reporting and grading system, which must be widely accepted and used in clinical practice.

To date, the Clavien-Dindo Classification (CDC) system still remains the main tool for the assessment of surgical complications. The CDC is a modified version of the original grading of complications published in 1992 as the T92 classification system; this was revised and validated in 2004 and was further evaluated in urological procedures by the European Association of Urology (EAU) in 2018.^3-5^ The CDC, however, does have limitations and attempts have been made to introduce new complication reporting and grading systems.

The aim of this study is to review the advancements in surgical complication classification systems and their validation, and further discuss their current and future roles in urological surgery.

## Classification Systems of Postoperative Complications

### Clavien-Dindo Classification System

In 1992, Clavien et al^3^ proposed the so-called T92 classification system to categorize complications of surgery based on the kind of interventions necessitated for treatment. This was critically re-evaluated and modified in 2004 to improve its accuracy, and the new grading system (CDC), which also relies on the therapy used to treat complications, is currently the most widely used for the assessment of perioperative morbidity and mortality.^4^ It classifies complications into 5 grades, containing 7 discrete ranks (1, 2, 3a/b, 4a/b, 5), based on increasing severity, with grade 1 standing for “any deviation from the standard” and grade 5 indicating patients’ death. The CDC is advantageous in terms of applicability and generalizability, categorizing complications based on the invasiveness of the therapy they require. The CDC system was re-evaluated by Clavien et al in 2009, utilizing complex clinical scenarios presented at the University of Zurich’s weekly morbidity and mortality (M and M) meetings.^6^ Adverse events were evaluated by surgeons from seven hospitals worldwide who reached a consensus agreement of more than 90% in their grading. The EAU recommends the systematic application of the CDC in Urology; however, the system certainly has limitations

A major drawback of CDC is that many patients experience more than one adverse event and, therefore, by reporting only the highest-grade complication, the system may underestimate the cumulative patient morbidity, thus leading to a loss of data. Due to the inherent characteristics of the data gathering process, significant adverse events have the potential to overshadow less significant but nonetheless noteworthy complications. Furthermore, the absence of a weighting system restricts cross-grade comparison.^7^ For example, the interpretation regarding the weighting of two complications of grade 2 with an additional CDC complication of grade 3 remains unclear. Moreover, the CDC only recognizes postoperative complications and provides no information on those occurring during surgery (Table 1).

### Memorial Sloan-Kettering Cancer Centre Secondary Events System

In 2001, the Department of Surgery at Memorial Sloan-Kettering Cancer Center (MSKCC) established the institutional Surgical Secondary Events (SSE) database to report any complications that arise within the first 30 days following surgery.^8^ Surgical Secondary Events is a modified version of the T92 classification system, as it assesses the consequences of an intervention or complication using a 5-grade scale. It also defines specific secondary events by 14 physiological categories, namely: cardiovascular system, infection, endocrine system, metabolism, gastrointestinal system, musculoskeletal system, general, nervous system, genitourinary system, pain, head and neck, pulmonary system, hematological or vascular system, wound, or skin. This categorization of adverse events seems to be the main advantage of MSKCC SSE over CDC. A main restriction of the MSKCC SSE system is that it does not record all grade 1 and 2 events. Nevertheless, it precisely records incidents that necessitate a modification in the patient’s degree of care, as well as subsequent occurrences that lead to irreversible damage or death of vital organs. Other limitations of this classification system are the fact that it focuses on cancer surgery and that it does not record adverse events beyond the 30th postoperative day.

A main difference between CDC and MSKCC SSE is the criteria used to determine grade 4 complications. In 2004, Dindo modified Clavien’s original classification scheme, expanding it from a 4- to a 5-grade system. The modification included the addition of a new grade 4 category, referring to life-threatening complications which require management in the intensive care unit (ICU). In contrast, grade 4 complications in the MSKCC SSE classification system are characterized by chronic disability or organ resection.

### Accordion Severity Grading System

The Accordion Severity Grading System, introduced in 2009, is based on CDC.^9^ It has been given this name “because of its ability to expand to accommodate the range of complications found in large complex studies while contracting for smaller studies.”^9^ It is capable of assessing a broad spectrum of complications that occur after surgery. The contracted classification has 4 levels: mild, moderate, and severe complications, and death due to a complication. The expanded classification has 6 levels: mild, moderate, severe with an invasive procedure without general anesthesia, severe with an operation under general anesthesia, severe with organ-system failure complications, and death. The expansion of the severe group into 3 subgroups was based on grade groups 3A, 3B, 4A, and 4B of the CDC. However, in the Accordion Severity Grading System, admission to the ICU is not a criterion for the severe group. It is also worth mentioning that the term “organ failure” has been strictly defined and is therefore easier to use. Despite the aforementioned strengths, the system has not been widely accepted and used by surgeons.

### Comprehensive Complication Index

As previously mentioned, the CDC has some drawbacks that cannot be overlooked. To overcome these weaknesses, Slankamenac et al^10^ proposed, in 2013, a new complication reporting system, the Comprehensive Complication Index (CCI). The CCI is based on the CDC but accounts for all accumulated complications and provides a continuous overall score between 0 and 100, with 100 indicating patient death. The ability of the CCI to assess adverse events longitudinally and provide cumulative patient morbidity is considered its major strength. However, when many complications occur, the overall score may exceed 100 even if the patient is alive, which appears to be a considerable limitation in the design of the CCI. To add to this, the CCI encompasses many of the drawbacks of the CDC, as it is based on it. A case in point would be complications of different morbidities categorized in a similar manner or different grading of the same complications as the same intervention may be done under general vs local anesthesia at different hospitals.

Over the last few years, CCI, which was first introduced and validated in General Surgery, has also been utilized in Urology.^11,12^ The clinical validation of CCI was done in major oncologic urological operations and in endourological procedures.^7,13-16^ Comprehensive Complication Index not only proved to be more accurate in assessing surgical complications but also reduced the required sample size for clinical trials.^7,16^ It was also shown to be better correlated with length of stay (LOS) when compared to CDC.^16,17^

In an attempt to improve some of the limitations of CCI, such as the pitfall of the overall score exceeding 100 in patients with many complications, Furrer et al^13^ introduced a modified version of CCI, the Bern CCI. The authors employed the Bern CCI to enhance the accuracy of short-term complication reporting following cystectomy and urinary diversion. It was originally designed to be urology-focused and significantly predicted the onset of death between postoperative days 91 and 365.^13^ The same researchers conducted an additional evaluation of the Bern CCI on patients who underwent open radical prostatectomy.^18^ The findings revealed that the Bern CCI offers a more accurate representation of postoperative morbidity compared to the original CCI. Hence, it justifies the need to explore implementing the Bern CCI as a uniform protocol for reporting complications that occur after major urological operations.

## Classification Systems of Intraoperative Complications

### Modified Satava Classification

In 2005, Satava proposed a simple approach for grading surgical errors during an operation.^19^ Using this approach, Kazaryan et al^20^ developed a 3-grade classification system for intraoperative incidents.^20^ This system can be applied to classify any type of surgery-related event that occurs during surgery (Table 2). Grade 1intraoperative incidents include those with no change to the operative approach and without further consequences for the patient; grade 2 incidents have further consequences for the patient, whereas grade 3 events are those with significant consequences for the patient. It is a quick and simple system that has been used in minimally invasive procedures, including ureteroscopy.^21,22^ Its main drawback is the possible misinterpretation of a grade due to the absence of a clear ranking in the 3-tier system.

### Intraoperative Adverse Incident Classification by EAU (EAUiaiC)

In 2020, an EAU ad hoc Complications Guidelines Panel demonstrated the EAUiaiC system.^23^ The categorization was created by a modified Delphi procedure, wherein 346 specialists responded to two rounds of survey questionnaires. Adverse incident terminology was assessed by experts using a 5-point Likert scale to measure clarity, comprehensiveness, hierarchical structure, mutual exclusivity, clinical usefulness, and quality enhancement. The grading system has eight degrees of adverse incidents, ranging from grade 0 (no deviation and no consequence to the patient) to grade 5B (wrong surgical site or intraoperative death). On the panel of experts, 125 of them deemed it highly favorable in terms of its pertinence and dependability. The EAUiaiC system has been used or validated in major urological procedures (radical cystectomy, radical and partial nephrectomy, radical prostatectomy), demonstrating good construct validity.^24-26^

### ClassIntra Classification

ClassIntra is a newly created and validated classification system used to grade intraoperative adverse events that transpire between the skin incision and skin closure.^27^ These events can originate from various sources, such as the surgery itself or anesthesia. ClassIntra was derived from the preexisting 2015 Classification of Intraoperative Complications (CLASSIC) system, which was developed by the same group after conducting a two-round Delphi study.^28^ In CLASSIC, adverse events are classified into four grades depending on the need for treatment (no need, grade 1; need for treatment, grade 2) and the severity of the complication (life-threatening or permanent disability, grade 3; death, grade 4). In order to conform to the validated CDC, the CLASSIC system was revised to incorporate five severity ratings, thus creating ClassIntra version 1.0. The decision criterion regarding the necessity of treatment and the severity of symptoms was retained. Due to its extensive practicability and exceptional dependability, ClassIntra has become the most frequently referenced intraoperative classification system. Nevertheless, there are limitations to consider, such as the possibility of reporting bias, which may arise from the outcome adjudicators not being blinded to intraoperative and postoperative adverse events. Additionally, there is the presence of potential confounding variables when evaluating LOS.

### Reporting Systems of Postoperative Complications

#### The Martin Criteria for Complication Evaluation

In 2002, Martin et al proposed a set of 10 standard criteria to ensure precise and thorough reporting of surgical complications. These criteria include aspects such as the techniques used to collect data, the length of follow-up, the definition of complications, the mortality and morbidity rate, the severity grade, and the duration of hospital stay (Table 3).^29^ The Martin criteria are considered to be the first reporting system for postoperative complications and have proved to be an effective protocol for the evaluation of patient’s burden. Furthermore, they have been used by urologists, as they report valuable outpatient information and identify risk factors associated with surgery, thus contributing significantly to the search for improving the quality of surgical care.^30,31^ The main limitation of this reporting system is the use of criteria with a broad scope, which affects the credibility of reporting.

### EAU Guidelines Panel Criteria

In 2012, an ad hoc EAU Guidelines Panel, based on previously published Martin criteria, issued a guideline consisting of 14 quality criteria necessary for precise and thorough reporting of outcomes following urological surgical procedures.^32^ According to the EAU working group, urologists should, when reporting complications, define the method of accruing data, define who collected the data, indicate the duration of follow-up, include outpatient information, include mortality data and causes of death, include definitions of complications, define procedure-specific complications, use a severity grading system (the CDC is recommended) and finally include risk factors, readmissions and their causes, reoperations, types and causes, and the percentage of patients lost to follow-up. The previous criteria were validated in 2016 in prostate cancer patients undergoing robotic-assisted radical prostatectomy in a single center.^33^ The application of the EAU Guidelines Panel criteria resulted in about a twofold increase in the complication rate following robot-assisted radical prostatectomy, compared to a retrospective analysis of patient charts. Furthermore, it facilitated the identification of post-discharge issues in over 15% of patients that would have otherwise gone unnoticed. Nevertheless, there has been a noticeable lack of compliance since inception.^34^

### Reporting Systems of Intraoperative Complications

#### ICARUS Global Surgical Collaboration Criteria

The ICARUS Global Surgical Collaboration criteria were introduced by Cacciamani et al. in 2022 in an attempt to standardize the assessment, reporting, and grading of intraoperative complications.^35^ The 13 ICARUS criteria were established using a modified Delphi technique with 35 surgeons. These criteria are relevant to all surgical specialties, including anesthesiology. Unlike the EAU Guidelines Panel criteria for postoperative complications, the ICARUS criteria are not yet validated.

## Conclusion

One can reasonably infer that each classification and reporting method possesses its own set of benefits and drawbacks. The CDC has been the primary system for reporting complications since its implementation due to its simplicity and versatility. However, because it only offers a general overview of surgical adverse events, new grading and reporting systems have been developed. This comprehensive review underlines the necessity of developing a new reporting and categorization system for complications that is specific to urology. This system should be comprehensive and replicable with validated accuracy and reliability. Ameliorating the complication reporting system is crucial for Urologists, as it holds the potential to enhance surgical outcomes in the future.

## Figures and Tables

**Table 1.** Classification Systems for Postoperative Complications

System	Brief Outline	Advantages	Drawbacks
Clavien-Dindo Classification (CDC) system	It relies on the therapy used to treat complications and classifies complications into 5 grades, containing 7 discrete ranks (1, 2, 3a/b, 4a/b, 5), based on increasing severity, with grade 1 standing for “any deviation from the standard” and grade 5 indicating patients‘ death.	High applicability and generalizability.	May underestimate the cumulative patient morbidity. No useful for cross-grade comparison.
Memorial Sloan-Kettering Cancer Centre Secondary Events (MSKCC SSE) system	It assesses the consequences of an intervention or complication using a 5-grade scale. It also defines specific secondary events by 14 physiological categories.	The categorization of adverse events by 14 physiological categories.	It does not record all grade 1 and 2 events. It focuses on cancer surgery and does not record adverse events beyond the 30th postoperative day.
Accordion Severity Grading System	The contracted classification has 4 levels: mild, moderate, and severe complications, and death due to a complication. The expanded classification has 6 levels: mild, moderate, severe with an invasive procedure without general anesthesia, severe with an operation under general anesthesia, severe with organ-system failure complications, and death.	It can assess a broad spectrum of complications.	Has not been widely accepted and used by surgeons.
Comprehensive Complication Index (CCI)	Accounts for all accumulated complications and provides a continuous overall score between 0 and 100, with 100 indicating patient death.	The ability of the CCI to assess adverse events longitudinally and to provide cumulative patient morbidity.	When many complications occur, the overall score may exceed 100 even if the patient is alive.

**Table 2.** Classification Systems of Intraoperative Complications

System	Brief Outline	Advantages	Drawbacks
Modified Satava classification	Grade 1 intraoperative incidents include those with no change to the operative approach and without further consequences for the patient; grade 2 incidents have further consequences for the patient, whereas grade 3 events are those with significant consequences for the patient.	Quick and simple system.	Possible misinterpretation of a grade due to the absence of a clear ranking in the 3-tier system.
Intraoperative Adverse Incident Classification by EAU (EAUiaiC)	The grading system has 8 degrees of adverse incidents, ranging from grade 0 (no deviation and no consequence to the patient) to grade 5B (wrong surgical site or intraoperative death).	High level of pertinence and dependability.	Use of scenarios derived from literature and expert opinions.
ClassIntra Classification	Classifies intraoperative adverse events that transpire between the skin incision and skin closure. Five severity grades depending on the need for treatment (no need, grade 1; need for treatment, grade 2) and the severity of the complication.	Extensive practicability and exceptional dependability.	Possibility of reporting bias, which may arise from the outcome adjudicators not being blinded to intraoperative and postoperative adverse events. Presence of potential confounding variables when evaluating length of stay.

**Table 3.** Reporting Systems of Postoperative/Intraoperative Complications

System	Brief Outline	Advantages	Drawbacks
The Martin criteria	A set of 10 standard criteria including aspects such as the techniques used to collect data, the length of follow-up, the definition of complications, the mortality and morbidity rate, the severity grade, and the duration of hospital stay.	Effective and widely used by Urologists.	Criteria with a broad scope, which affects the credibility of reporting.
EAU Guidelines Panel criteria	Fourteen quality criteria necessary for precise and thorough reporting of outcomes following urological surgical procedures, based on previously published Martin criteria.	Validated in prostate cancer patients undergoing robotic-assisted radical prostatectomy. They facilitate the identification of post-discharge complications.	Lack of compliance since inception.
ICARUS Global Surgical Collaboration Criteria	The 13 ICARUS criteria were established using a modified Delphi technique with 35 surgeons and are relevant to all surgical specialties, including anesthesiology.	Comprehensive criteria that are relevant to all surgical specialties, including anesthesiology.	Not yet validated.
